# Predictors of dental visits among primary school children in the rural Australian community of Lithgow

**DOI:** 10.1186/s12913-017-2232-1

**Published:** 2017-04-11

**Authors:** James Rufus John, Haider Mannan, Subrat Nargundkar, Mario D’Souza, Loc Giang Do, Amit Arora

**Affiliations:** 1 School of Science and Health, Western Sydney University, Campbelltown, NSW Australia; 2 Centre for Health Research, School of Medicine, Western Sydney University, Campbelltown, NSW Australia; 3 Clinical Research Centre, Sydney Local Health District, Camperdown, NSW Australia; 4 Australian Research Centre for Population Oral Health, University of Adelaide, Adelaide, SA Australia; 5 Oral Health Services and Sydney Dental Hospital, Sydney Local Health District, Surry Hills, NSW Australia; 6 Discipline of Paediatrics and Child Health, Sydney Medical School, Westmead, NSW Australia; 7 Collaboration for Oral Health Outcomes Research, Translation, and Evaluation (COHORTE) Research Group, Ingham Institute for Applied Medical Research, Liverpool, NSW Australia

**Keywords:** Dental visit, School children, Rural, Socio-demographics, Oral health

## Abstract

**Background:**

Regular dental attendance is significant in maintaining and improving children’s oral health and well-being. This study aims to determine the factors that predict and influence dental visits in primary school children residing in the rural community of Lithgow, New South Wales (NSW), Australia.

**Methods:**

All six primary schools of Lithgow were approached to participate in a cross-sectional survey prior to implementing water fluoridation in 2014. Children aged 6–13 years (*n* = 667) were clinically examined for their oral health status and parents were requested to complete a questionnaire on fluoride history, diet, last dental visit, and socio-demographic characteristics. Multiple logistic regression analyses were employed to examine the independent predictors of a 6-monthly and a yearly dental visit.

**Results:**

Overall, 53% of children visited a dentist within six months and 77% within twelve months. In multiple logistic regression analyses, age of the child and private health insurance coverage were significantly associated with both 6-monthly and twelve-month dental visits. In addition, each serve of chocolate consumption was significantly associated with a 27% higher odds (OR = 1.27, 95% CI: 1.05-1.54) of a 6-monthly dental visit.

**Conclusion:**

It is imperative that the socio-demographic and dietary factors that influence child oral health must be effectively addressed when developing the oral health promotion policies to ensure better oral health outcomes.

## Background

Dental caries is one of the most prevalent childhood chronic diseases worldwide, which demands the need for an appropriate public health response [1]. In the Australian context, the Australian Institute of Health and Welfare (AIHW) reported that over 50% of Australian children aged 6 to 12 years had caries experience either in their primary or permanent teeth [2]. Numerous studies have reported that untreated dental caries in children strongly influences their growth, cognitive development and quality of life, thereby leading to failure to thrive in severe untreated cases [3, 4]. Whilst there are various negative health ramifications associated with dental caries in children, it is fortunate that the disease is largely preventable with proper oral hygiene, diet with less sugar content, ideal fluoride levels and regular dental visits [5].

Frequency of dental visits is one of the important factors that contributes to an individual’s oral health and well-being. Studies report that regular dental attendance in childhood not only sets positive health behaviour and a healthy trajectory for the long run, but also significantly improves the quality of life [6, 7]. Langevin et al. [7] claimed that regular dental visits increase the probability of diagnosing and managing oral diseases in their early stages, thereby limiting any significant or irreversible damage to teeth and gums. Given the scarcity of robust scientific evidence on the appropriate frequency of dental visits, the Australian Research Centre for Population Oral Health (ARCPOH) [8, 9] recommends that frequency must be based on different oral health needs and individual risk levels. The AIHW [10] reports that 69.4% of Australian children and teenagers visited the dentist within the past year whereas a significant proportion of 16.9% failed to visit in the last 5 years.

Recent statistical reports also state that the pattern of overall caries experience in terms of mean dmft/DMFT (decayed, missing and filled teeth) scores has gradually risen from 1.45 in 1996 to 2.58 in 2010 among children aged 6 years and from 0.84 in 2000 to 1.34 in 2010 in children aged 12 years [2]. In addition, it is seen that the caries distribution is strongly influenced by the disparity in oral health outcomes among Australian children as a result of parental attitude, affordability and access to dental services [10]. For example, AIHW [10] reported that Australian children belonging to lower socio-economic status (SES) groups had fewer visits on average compared to the high SES counterparts (77.5 and 55.2% respectively). In addition, the report also states that Australia children residing in regional to remote areas had fewer annual dental visits on average compared to children living in major cities.

Lithgow Local Government Area (LGA) is a rural community situated 145 km west of Sydney with a population of 19,756 people. In 2012, Lithgow LGA was the only community with a non-fluoridation status within the confines of the former Sydney West Area Health Services [11]. Based on fluoridation and caries risk status, Arora and Evans [12] reported that caries prevalence among children in the non-fluoridated community of Lithgow was notably higher than children residing in the fluoridated communities of Bathurst and Orange. In addition, Lee & Brearley-Messer [13] reported that children aged 5–6 years and 11–12 years, living in non-fluoridated or low-fluoridated communities had 60 and 42% higher dmft/DMFT (decayed, missing, filled teeth index) scores respectively compared to those residing in fluoridated areas. Thus, oral health status and dental visits in children shows a significant association to the remoteness of residence, where poor attendance, limited fluoride exposure and poor oral health was observed among children living in regional communities compared to urban residences [14].

**Fig. 1:**
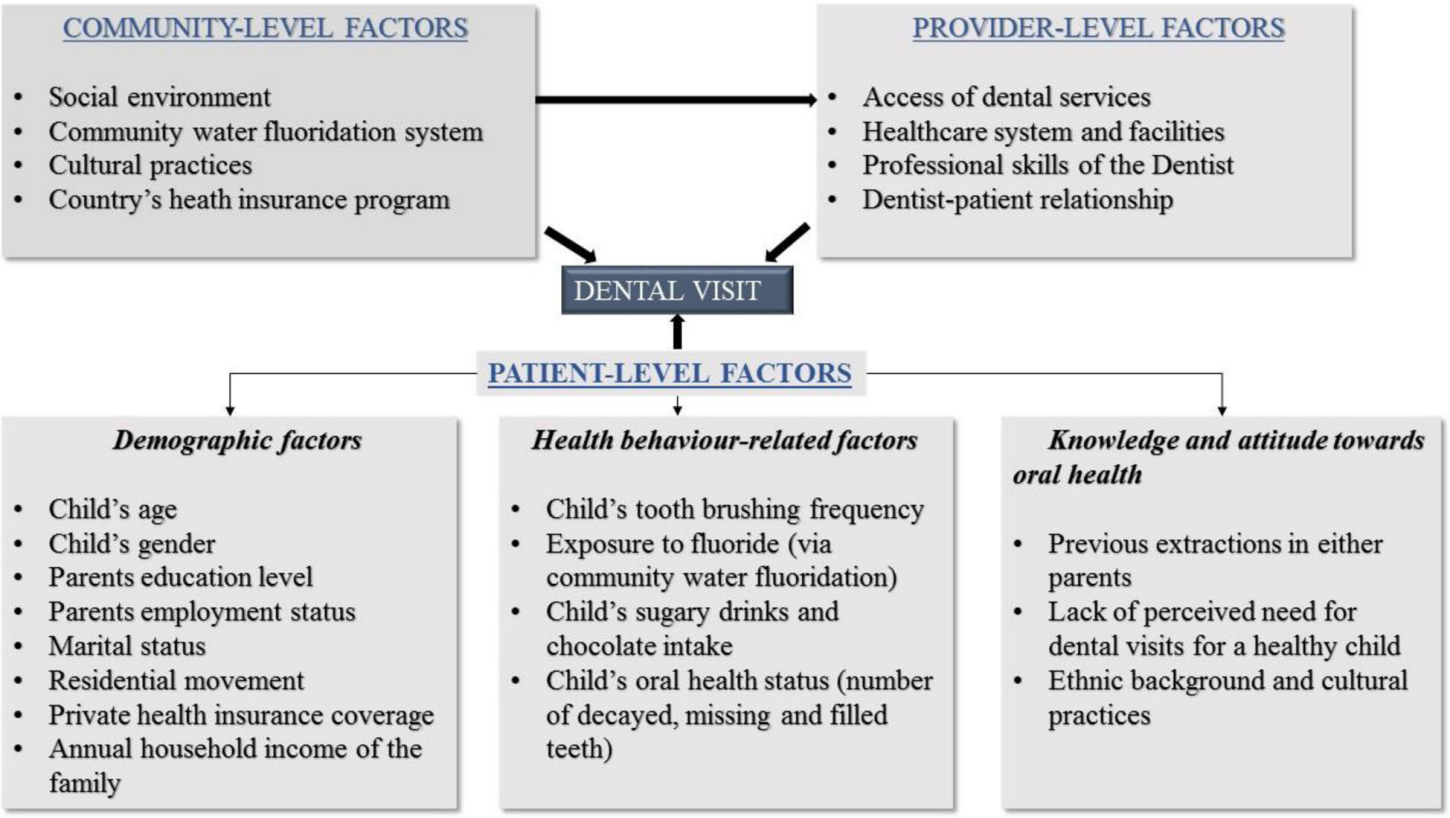
Conceptual model showing the influence of community-level, provider-level and patient-level factors on regular dental visiting

Figure 1 shows a theoretical model that summarises a triad of community-level, provider-level and patient-level factors that influence regular dental visit behaviours in children, based on Badri et al’s systematic review and Fisher-Owens’ model [15, 16]. These include community-level factors such as social environment, cultural practices and health insurance coverage; Provider-level factors such as access to dental services, professional skills of the dental team, and patient-doctor relationship; and Patient-level factors such as child’s age, gender and health behaviours; parent’s education, marital status, socio-economic status and the prioritisation of dental visits. Gussy et al. [17] conducted a survey among parents in rural Victoria and found that parental attitudes and behaviours influenced their children’s dental visit and oral health status. Besides the study by Gussy and colleagues, the literature on the factors influencing dental visits among Australian rural communities is scarce. Therefore, this study aims to determine the factors that influence dental visits in primary school children living in the rural community of Lithgow, Australia.

## Methods

### Population of interest and sampling

This study is a secondary data analysis of a cross-sectional survey conducted among primary school children in Lithgow LGA of New South Wales, prior to having access to water fluoridation in 2014 [11]. A letter of invitation was sent to all the six primary school principals of the Lithgow LGA to participate in the survey. The parents of the primary school children were then approached to partake in the survey and were provided with a take-home information pack containing a detailed oral health questionnaire and a consent form. In addition, weekly reminders about the clinical examination were sent to the parents for 4 weeks using the school newsletters.

### Questionnaire survey

The outline of the dental research questionnaire was aligned closely to the National Child Oral Health Survey questionnaire to allow standardised data collection and for comparison with the national census reports [18]. The questionnaire comprised of a detailed briefing on the child’s age, gender, brushing and dietary patterns, residency and their previous dental visit. In addition, the socio-demographic characteristics of the children’s parents such as age, information on their country of birth, Indigenous background, private health insurance coverage, tooth extraction history and annual household income were collected to analyse if ethnicity played a role in their children’s dental visits. The dietary questions focused on the number of serves of chocolates and carbonated drinks, fruit juices and cordial drinks consumed by the child on a usual day.

The tooth brushing frequency was categorised as children brushing with a fluoridated toothpaste once or less and twice or more daily. The number of decayed, missing and filled teeth (dmft) scores were dichotomised as no dmft scores and one or more dmft scores. Parents’ education was categorised as tertiary education and high school level education or less. In terms of employment, the three categories included managers and professionals, skilled workers and pensioners and unemployed.

### Clinical examination

After receiving the parents’ written consent, children were clinically examined in the school premises using a halogen light source. The guidelines of the World Health Organisation (WHO) were adopted as the diagnostic criteria for dental caries defining a carious tooth as a cavity into the surface of dentine [19]. All the patients’ teeth were examined wet, with a ball-ended WHO probe which was employed for examination if necessary [19]. The commonly used indices such as dmft index (for primary dentition) and DMFT index (for permanent dentition) were used and caries prevalence was denoted by the mean number of teeth that were decayed, missing (as a result of decay by extraction) and filled (because of decay) [20].

### Data quality

The clinical scores of the primary examiner were calibrated daily under the supervision of a calibrating examiner, and examiner reliability was assessed via Cohen’s Kappa statistic [21] on duplicated scores obtained from both intra- and inter-examinations. The kappa value for inter-examiner reliability was 0.93, and the intra-examiner reliability was 0.98. The questionnaire data was entered twice and inconsistencies were removed by cross-checking the questionnaire.

#### Data analysis

All the statistical analysis for this study was conducted with SPSS version 24. Descriptive characteristics were calculated using crosstabs to obtain the row percentages of each variable in relation to the dental visits in the last 6 months. Multiple logistic regression analyses were performed to determine the association between the factors and the two dichotomous outcome variables of dental visits in the last 6-months and twelve months. In addition, the presence of influential outliers for each variable was determined and analysed using difference of fit [22]. There was approximately 8% missing data in the study sample which was managed by employing the multiple imputation analysis. Multiple imputation makes repeated draws from the model of distribution of variables having missing data and provides valid values using other available information from the dataset [23, 24]. Missing values were imputed 25 times using a Markov Chain Monte Carlo (MCMC) algorithm known as fully conditional specification (FCS) or chained equations to reduce the effect of sampling variability in the parameter estimates [25]. The outcome variable of dental visits was included as a covariate in the imputation model, as it is recommended that the inclusion of the dependent variable of the risk prediction model in the imputation model enables unbiased estimates of model coefficients [26].

All variables present in the theoretical model (Fig. 1) were fitted in the multiple logistic regression analyses to determine the factors that were independent predictors of dental visit in Lithgow school children. Variables that had a non-significant effect on the model were sequentially eliminated in a backward stepwise manner. All variables in the final model were variables for which, when excluded, the change in deviance compared with the corresponding Chi-square (*Χ* ^2^) test statistic on the relevant degrees of freedom was significant (p < 0.05). In addition, all the variables in the conceptual model were tested for collinearity against each other and against other covariates, before including them in multiple logistic regression analysis. However, all variables tested had pairwise correlations of less than 0.5 implying that the possibility of collinearity between the variables is small.

A total of six models were examined in the study to explore all the different possible outcomes with the 6-monthly and yearly visit variables, and to observe their significance associated to each model. Models 1 to 4 were analysed for original data without correction of missing observations while Models 5 and 6 incorporated the imputed data. Whilst model 1, 2 and 5 have six monthly visit as the outcome; model 3, 4 and 6 have yearly visit as the outcome variable. To examine the external validity of the results, the observed population estimate of the Lithgow survey was compared against the 2011 ABS census [27] by performing one sample z-tests for proportions.

## Results

A total of 1589 parents were approached for the Lithgow LGA survey. Of these, 667 (42%) completed the questionnaire survey and gave consent for their children to be clinically examined. It was noted that 53% of children visited the dentist within six months and 77% within in the last year.

Table 1 shows the descriptive statistics of the frequency distribution of the socio-demographic and health-behavioural factors with the outcome variable of 6-monthly dental visits. In addition, median scores were calculated when the Shapiro-Wilk test indicated that the continuous variables that had significant deviation from the normal distribution. Table 2 shows the predictors that are significantly associated with dental visits at 6-month and twelve-month period using the original and non-imputed data whereas the statistically significant predictors for dental visits using imputed data are shown in Table 3.

**Table 1:** Descriptive statistics of socio-demographic and health-behavioural factors influencing 6-monthly dental visit in Lithgow school children

Factors influencing dental visits.	*N*	Has the child visited the dentist in the last 6 months?		Chi-squared *p*-value
		Yes *n* =353	No *n* = 312	
Age of the child (In years - MAD)	665	353 (9.0)	312 (8.0)	0.278
Gender of the child	665			0.921
Male		179 (50.7%)	157 (49.3%)	
Female		174 (50.3%)	155 (49.7%)	
Frequency of tooth brushing	663			0.001*
Once or less		110 (31.3%)	135 (43.4%)	
Twice or more		242 (68.8%)	176 (56.6%)	
Decayed, missing and filled teeth status	665			0.025*
No dmft/DMFT		156 (44.2%)	165 (52.9%)	
One or more dmft/DMFT		197 (55.8%)	147 (47.1%)	
Has the child always lived in Lithgow?	665			0.009*
Yes		233 (66.0%)	120 (34.0%)	
No		175 (56.1%)	137 (43.9%)	
Number of Serves of sugar sweetened beverages consumed per day (MAD)	654	346 (2.0)	307 (3.0)	0.131
Number of serves of chocolate per day (MAD)	638	337 (1.0)	301 (1.0)	0.091
Child in single parent household?	665			0.277
Yes		65 (18.4%)	68 (21.8%)	
No		288 (81.6%)	244 (78.2%)	
Age of the Mother (In years – MAD)	659	348 (37.0)	311 (36.0)	0.040*
Age of the Father^a^ (In years – MAD)	527	283 (39.0)	244 (38.0)	0.003*
Mother’s country of birth	653			0.949
Overseas		29 (8.4%)	26 (8.5%)	
Australia		318 (91.6%)	280 (91.5%)	
Father’s country of birth^a^	529			0.754
Overseas		20 (7.0%)	19 (7.8%)	
Australia		264 (93%)	226 (92.2%)	
Mother’s Indigenous status	652			0.028*
Indigenous		5 (1.4%)	13 (4.3%)	
Non-Indigenous		342 (98.6%)	292 (95.7%)	
Father’s Indigenous status^a^	535			0.415
Indigenous		5 (1.8%)	7 (2.8%)	
Non- Indigenous		280 (98.2%)	243 (97.2%)	
Education level of the Mother	651			0.297
University or College		91 (26.2%)	69 (22.7%)	
High school or vocational training		256 (73.8%)	235 (77.3%)	
Education level of the Father^a^	530			0.360
University or College		46 (16.3%)	48 (19.4%)	
High school or vocational training		236 (83.7%)	200 (80.6%)	
Mother’s employment	654			0.006*
Managers and professionals		75 (21.5%)	63 (20.7%)	
Skilled workers		173 (49.6%)	119 (39%)	
Pensioners and unemployed		101 (28.9%)	123 (40.3%)	
Father’s employment^a^	526			0.490
Managers and professionals		88 (31%)	74 (30.6%)	
Skilled workers		179 (63%)	147 (60.7%)	
Pensioners and unemployed		17 (6%)	21 (8.7%)	
Number of tooth extractions in Mother (MAD)	665	353 (0.0)	312 (1.0)	0.139
Number of tooth extractions in Father^a^ (MAD)	536	286 (0.0)	250 (2.0)	0.051
Private health insurance coverage	628			<0.001*
Yes		161 (48.3%)	81 (28.7%)	
No		172 (51.7%)	214 (72.5%)	
Annual household income	497			<0.001*
High (over 100,000)		42 (16.8%)	28 (11.3%)	
Medium (40,000-100,000)		133 (53.2%)	101 (4.9%)	
Low (up to 40,000)		75 (30.0%)	118 (47.8%)	

^a^Lower number of observations noticed in relation to father’s characteristics such as age, country of birth, Indigenous status, education, employment and extraction correspond to the children residing in a single parent household with mothers, where fathers’ details are not applicable

MAD - Only median values were calculated for all continuous covariates because Shapiro-Wilk tests showed that they significantly deviated from following the Normal distribution

*Variables found to be statistically significant (*p* < 0.05) using Pearson’s chi-squared test

**Table 2:** Multiple logistic regression of individual and combined^a^ parental characteristics with 6-monthly and twelve-monthly dental visits

Variables	Model 1 (N = 585)	Model 2 (N = 590)	Model 3 (N = 585)	Model 4 (N = 589)
	Adjusted OR with 95% CI^b^	Adjusted OR with 95% CI^b^	Adjusted OR with 95% CI^b^	Adjusted OR with 95% CI^b^
Age of the subject	0.92 (0.85, 0.99)*	0.92 (0.85, 0.99)*	0.87 (0.79, 0.96)*	0.88 (0.80, 0.97)*
Decayed, missing and filled teeth Status	NS^2^	NS^2^		
No dmft/DMFT score			1.00	1.00
One or more dmft/DMFT score			0.64 (0.43, 0.94)*	0.62 (0.42, 0.92)*
Serves of chocolate per day	1.27 (1.05, 1.54)*	1.27 (1.05, 1.54)*	NS^2^	NS^2^
Education level of the Mother	NS^2^	NS^2^		NS^2^
University or College			1.00	
High school or vocational training			2.28 (1.34, 3.91)*	
Highest employment in the household	NS^2^	NS^2^	NS^2^	
Managers and professionals				1.00
Skilled workers				1.47 (0.69, 3.15)*
Pensioners and unemployed				2.64 (1.21, 5.75)*
Private health insurance coverage				
Yes	1.00	1.00	1.00	1.00
No	0.43 (0.30, 0.60)*	0.43 (0.30, 0.60)*	0.54 (0.35, 0.83)*	0.62 (0.39, 0.97)*

Model 1 - Original data with outcome variable as 6-month visit and individual parental characteristics

Model 2 – Original data with outcome variable as 6-month visit and combined parental characteristics

Model 3 – Original data with outcome variable as yearly visit and individual parental characteristics

Model 4 - Original data with outcome variable as yearly visit and combined parental characteristics

^a^Combined parental traits indicate the highest of either parents’ factors such as education, employment and extraction history

*OR* Odds ratio

^b^Confidence interval

*Statistically significant (*p*-value < 0.05)

NS^2^ Non-significant (*p*-value > 0.05)

**Table 3:** Multiple logistic regression of imputed data comparing individual parental characteristics (6-month and 12-monthly dental visits)

Variables	Model 5 (*N* = 665)	Model 6 (*N* = 665)
	Adjusted OR with 95% CI^a^	Adjusted OR with 95% CI^a^
Age of the subject	0.91 (0.85, 0.99)*	0.87 (0.80, 0.96)*
Decayed, missing and filled teeth status	NS^2^	
No dmft/DMFT scores		1.00
One or more dmft/DMFT scores		0.60 (0.42, 0.88)*
Serves of chocolate per day	1.25 (1.03, 1.52)*	NS^2^
Education level of the Mother	NS^2^	
University or College		1.00
High school or vocational training		2.02 (1.23, 3.32)*
Extraction history of Mother	1.04 (1.00, 1.08)*	NS^2^
Private health insurance coverage		
Yes	1.00	1.00
No	0.46 (0.34, 0.64)*	0.54 (0.36, 0.80)*

Model 5 - Imputed data with outcome variable as 6-month visit and individual parental characteristics

Model 6 - Imputed data with outcome variable as yearly visit and individual parental characteristics

*OR* Odds ratio

^a^Confidence interval

*Statistically significant (*p*-value < 0.05)

NS^2^ Non-significant (*p*-value > 0.05)

Predictors such as age of the child and private health insurance coverage were statistically significant (*p*-value ≤ 0.05) against both the outcome variables of dental visits (6-month and yearly visits) in all models as seen in Tables 2 and 3. In terms of age as a predictor variable, every year of increased age was associated with 8% lower odds (OR = 0.92, 95% CI: 0.85-0.99) (Table 2) in the 6-month visit and 13% lower odds in the yearly dental visit respectively (OR = 0.87, 95% CI: 0.79-0.96) (Table 2). Similarly, it is noted that children who had private health insurance had a 57% lower odds for a 6-month dental visit (OR = 0.43, 95% CI: 0.30-0.60) and a 46% lower odds for an annual dental visit (OR = 0.54, 95% CI: 0.35-0.83) (Table 2). Furthermore, each additional serve of chocolate consumption increased children’s odds of visits the dentist in the last six months by 27% (OR = 1.27, 95% CI: 1.05-1.54) (Table 2).

Compared to mothers who have a university or college degree, mothers who went to high school or completed a vocational degree had about twice the odds of taking their child for a dental visit in the last 12 months (OR = 2.28, 95% CI: 1.34-3.90) (Table 2). However, in the combined household model of annual visit (Table 2), pensioners and unemployed parents had more than twice the odds of taking their children to the dentist compared to the children born to managers and professional parents (OR = 2.64, 95% CI: 1.21-5.75). Finally, children having one or more dmft/DMFT scores had 37% lower odds of an annual dental attendance compared to children who do not have any dmft/DMFT scores (OR = 0.63, 95% CI: 0.43-0.94) (Table 2).

In terms of the key differences between the non-imputed and imputed models, Table 3 shows that mother’s extraction history had significant association with dental visits at 6-monthly period in the imputed model (OR = 1.04, 95% CI: 1.00-1.08). Besides mothers’ extraction history, no major differences were observed between the non-imputed and imputed models. On the contrary, the variable relating the children’s previous exposure to fluoridated water was found to be non-significant in the multiple analysis.

## Discussion

This study provides a snapshot of the socio-behavioural and health-behavioural determinants on regular dental visits in primary school children living in the rural community of Lithgow, Australia. Overall, 53% of children visited a dentist within six months and 77% within twelve months. In multiple logistic regression analyses, age of the child and private health insurance coverage were significantly associated with both 6-monthly and twelve-month dental visits. In addition, each serve of chocolate consumption was significantly associated with a 27% higher odds (OR = 1.27, 95% CI: 1.05-1.54) of a 6-monthly dental visit.

Numerous studies have reported that children living in a single parent household have higher odds of having poor oral health than children living in two parent households [28, 29]. In this study, one in five children were living in a single parent household with mothers, which stands out as an independent predictor for dental visits. The multiple regression analyses further show significantly lower odds of dental visits (for both outcomes) with increasing age of the child and private health insurance coverage which are consistent across all the six models (Tables 2 and 3). It is noted that children aged 6 to 7 years would experience loss of some primary teeth and eruption of first permanent molars which would be uncomfortable [30]. Therefore, children with perceived needs (perception of parents) are more likely to be taken for regular dental visits to assess their eruption patterns and their overall oral health status [30]. In addition, maternal dental anxiety and concern about their child’s untreated caries and oral health are also reported to be significant predictors for regular dental service utilisation in children [31].

Children who were covered by private health insurance had lower odds of dental visits within the last 6 and 12 months of the survey (Table 2). This is consistent with AIHW findings which reported that uninsured children had a problem-oriented dental visits pattern and were more likely to visit the dentist for symptomatic relief compared to insured children [10]. In addition, evidence also suggests that nearly half the children attending public dental service did not have private health insurance [10].

Unsurprisingly, there was a statistically significant association between serves of chocolate and the increase in the odds of dental visits (6-monthly). Numerous studies have reported that higher chocolate consumption leads to more numbers of decayed teeth which eventually needs to be restored [32, 33]. The key difference in the imputed model was that extraction history of the mother was also significantly associated with 6-monthly dental visits as seen in Table 3. This is consistent with the findings of Dye et al. [34] who reported that children born to mothers who had high rates of tooth loss had three times higher odds of having poor oral health outcomes. Therefore, it is evident that poorer maternal oral health is associated with substandard child oral health due to poor health behaviours and lifestyle choices [34].

Interestingly, children of mothers who did not have a university degree were more likely to visit the dentist annually. This finding contradicts most of the studies which report that mothers having higher education level have higher odds of taking their children for regular dental visits and vice versa [35, 36]. However, mothers with low education level have less oral health knowledge and are likely to have their child visit the dentist for an interventional treatment rather than a regular dental check-up [36]. However similar studies have reported that children of parents with low education levels visited a dentist more frequently (2.7 times) than children of highly educated parents for symptomatic relief [37]. Furthermore, Moimaz et al. reported that the greatest need for oral health treatment belonged to children of mothers with low education [38].

In this study, it is seen that children with one or more dmft/DMFT scores had lower odds of making an annual dental visit. It is anticipated that this is due to the fact that children living in rural communities visit the dental visits only for symptomatic relief [39]. In addition, pensioners and unemployed parents in the study had twice higher odds to take their child for an annual dental visit. This may be because lower socio-economic residents in NSW are provided with a concession card which enables their family to access free public oral health services [10]. Further, the AIHW reports that children of parents who were cardholders, had twice higher odds of attending a public dental care annually especially for symptomatic relief compared to non-cardholders [10].

In this study, 98% of the children in the study reported to use fluoridated toothpaste. Numerous studies [40, 41] have established the invaluable clinical significance of fluoridated toothpaste and its beneficial effects on teeth. However, in the current study the use of fluoridated toothpaste was not statistically significant to dental visiting.

Although a response rate of 50% is considered reasonable for validity of a study, lower response rates do not necessarily result in bias [42]. The Lithgow survey had a response rate of 42% which was less than anticipated. It was expected that response behaviour should have been higher because of the weekly reminder notices posted in the school newsletters [43]. In addition, low response rates might have been due to lack of interest in participating in surveys that are not perceived as a salient need in a person’s life or due to the proliferating health literacies previously published by other health research articles [44, 45].

In order to clarify the potential for bias due to low response rates and to establish the generalisability of the study, the observed percentages of categories for selected socio-demographic variables from Lithgow survey were compared to the corresponding expected percentages of the same variables based on the selected postcodes of Lithgow from the 2011 ABS census reports as seen in Table 4 [27]. Three socio-demographic factors such as household country of birth, Indigenous status and education level were considered for comparison. The comparison demonstrated that the household education level and Indigenous status as estimated by Lithgow survey did not differ significantly from the corresponding variables for Lithgow based on census data. The study sample overestimated the proportion of children born to Australian parents as compared to the Census by 3%. However, this overestimation is not expected to influence the study’s outcomes because parent country of birth was not significantly associated with the primary outcome of the study (Table 1).

**Table 4:** Population benchmark comparison of demographic characteristics of Lithgow from ABS census 2011 report

Socio-demographic characteristics	Survey estimate (observed percentages) % of children (95% CI)	Observed *p*-value^ӂ^	2011 census report (expected percentages) % of children
Country of birth of household^a^		0.002*	
Overseas	11.5 (9.07-13.93)		16.45
Australia	88.5 (86.07-90.9)		83.55
Indigenous status of household^b^		0.079	
Indigenous	4.38 (2.82-5.94)		5.57
Non-Indigenous	95.62 (94.06-97.18)		94.43
Highest education level in the household ^c^		0.267	
University or College degree	28.74 (25.29-32.19)		26.83
High school or vocational training	71.26 (67.81-74.71)		73.17

^ӂ^ Added *p*-value was obtained from z-test

*Statistically significant at 5 percent level

^a^Children were classified to the overseas born category if they had at least one parent who was born overseas

^b^Children were classified to the Indigenous category if they had at least one parent who was Indigenous

^c^Children were classified to the University or College degree category if they had at least one parent who had a university or college degree

In terms of the strengths of the study, the various possible combinations with outcome variables of 6-month and yearly dental visits were analysed and examined. Although there were only a few missing observations, multiple imputations were employed to obtain complete cases which were compared to the corresponding original models. In addition, this study also provides valuable information on the drivers and barriers of regular dental visits in Lithgow children which could prove useful in policy development. However, this study also has some opportunities for improvement worth reporting. This study used the WHO criteria to examine caries for field or epidemiological surveys. That is, caries in dentine (obvious cavitation seen with naked eye) was recorded on wet tooth surfaces. A drawback of the WHO method is that caries in the enamel cannot be examined which often requires proper dental equipment to isolate or dry the teeth. The low response rate and the use of self-reported questionnaire might have contributed to some degree of bias affecting the results [46]. Although the questionnaire had good detail of all the factors, the reason for previous visit (six months or twelve months) was not recorded. Furthermore, there is difficulty in establishing causation using the cross-sectional study design. In terms of scope for future research, the information of Lithgow survey could be used to possibly compare the oral health status before and after the effect of fluoridation. In addition, further research is needed to explore the impact of other possible predictors such as the role of dental phobia and anxiety on regular dental visits in children, and the parental perception on the level of prioritization of oral health for their children.

## Conclusion

This study provides insight on the impacts of various social determinants on regular dental visits among primary school children living in the rural community of Lithgow, Australia. On the other hand, the utilisation of dental services and patterns of use also serve as critical indicators of oral health-related beliefs and behaviours of parents. It is imperative that the facilitators and barriers of regular dental visits in children residing in the regional Australian communities must be effectively addressed when developing the oral health promotion policies to ensure better health outcomes.

## Abbreviations

ABS: Australian Bureau of Statistics
AIHW: Australian Institute of Health and Welfare
ARCPOH: Australian Research Centre for Population Oral Health
CI: Confidence intervals
DF: Difference of fit
DMFT: Decayed, missing and filled teeth in permanent dentition
dmft: Decayed, missing and filled teeth in primary dentition
FCS: Fully conditional specification
LGA: Lithgow Government Area
MCMC: Markov Chain Monte Carlo
NHMRC: National Health and Medical Research Council
NS: Non-Significant
NSW: New South Wales
OR: Odds ratio
SES: Socio economic status
SPSS: Statistical package for the social sciences
WHO: World Health Organisation
